# Liver Stiffness by Transient Elastography Correlates With Degree of Portal Hypertension in Common Variable Immunodeficiency Patients With Nodular Regenerative Hyperplasia

**DOI:** 10.3389/fimmu.2022.864550

**Published:** 2022-05-06

**Authors:** Daniel V. DiGiacomo, Jessica E. Shay, Rory Crotty, Nancy Yang, Patricia Bloom, Kathleen Corey, Sara Barmettler, Jocelyn R. Farmer

**Affiliations:** ^1^ Department of Medicine, Division of Rheumatology, Allergy and Immunology, Massachusetts General Hospital, Boston, MA, United States; ^2^ Department of Medicine, Division of Gastroenterology, Massachusetts General Hospital, Boston, MA, United States; ^3^ Department of Pathology, Massachusetts General Hospital, Boston, MA, United States; ^4^ Department of Medicine, Division of Gastroenterology, University of Michigan, Ann Arbor, MI, United States

**Keywords:** common variable immunodeficiency (CVID), nodular regenerative hyperplasia (NRH), transient elastography (TE), fibroscan©, liver disease, liver biopsy

## Abstract

Nodular regenerative hyperplasia (NRH) is associated with high morbidity and mortality in patients with common variable immunodeficiency (CVID). While liver biopsy is the gold standard for NRH diagnosis, a non-invasive technique could facilitate early disease recognition, monitoring, and/or immune intervention. We performed a cross-sectional analysis of ultrasound-based transient elastography (TE) in patients with CVID to evaluate liver stiffness and compared this between patients with (N = 12) and without (N = 6) biopsy-proven NRH. Additionally, these data were compared to a cohort followed at our institution for non-alcoholic fatty liver disease (NAFLD) (N = 527), a disease for which TE has routine diagnostic use. Clinical and pathologic features of NRH were evaluated as correlates of liver stiffness, and receiver operating characteristic curves were used to define a liver stiffness cutoff with diagnostic utility for NRH among CVID patients. CVID patients with NRH had a more severe disease presentation compared to those without. This included increased autoinflammatory disease comorbidities, combined B-cell and T-cell dysfunction, and abnormal liver biochemistries (specifically an increased mean alkaline phosphatase level [proximal to TE, 250 vs. 100 U/L; p = 0.03; peak, 314 vs. 114 U/L; p = 0.02). Results of TE demonstrated a significantly elevated liver stiffness in CVID patients with NRH (mean 13.2 ± 6.2 kPa) as compared to both CVID patients without NRH (mean 4.6 ± 0.9 kPa) and non-CVID patients with NAFLD (mean 6.9 ± 5.5 kPa) (p < 0.01). No single or composite histopathologic feature of NRH correlated with liver stiffness including nodule size, nodule density, sinusoidal dilation, fibrosis, and/or lymphocytosis. In contrast, liver stiffness by TE was significantly correlated with clinical parameters of portal hypertension, including an elevated hepatic venous pressure gradient, an increased splenic longitudinal diameter, presence of varices, and presence of peripheral edema. A liver stiffness of greater than or equal to 6.2 kPa was a clinically significant cutoff for NRH in CVID patients. We propose that TE has diagnostic utility in CVID, particularly in the presence of immunophenotypic features such as combined B-cell and T-cell dysfunction, autoinflammatory comorbidities, and/or abnormal liver tests. Elevated liver stiffness by TE should raise suspicion for NRH in patients with CVID and prompt expedited evaluation by hepatology.

## Introduction

Common variable immunodeficiency (CVID) is the most frequent symptomatic antibody deficiency in adults (1). It comprises a heterogeneous group of disorders with increased infectious risk resulting from impaired and dysregulated immunity. Diagnosis requires low immunoglobulin (Ig) G combined with low IgA or IgM, impaired vaccine response, and the exclusion of secondary causes. While patients typically present with recurrent infections, more than 30% additionally demonstrate non-infectious manifestations (2). Liver disease is a frequent and underrecognized complication in patients with CVID and can be a consequence of recurrent infectious insults, malignancy, and/or immune dysregulation impacting the liver. The most prevalent liver pathology in patients with CVID is nodular regenerative hyperplasia (NRH), which affects 41%–87% of patients with CVID who undergo liver biopsy (3–6). NRH is believed to be mediated through T-cell infiltration of the sinusoidal endothelium, causing intra-hepatic vasculopathy. This process leads to hepatocyte damage, regeneration, and the characteristic nodular appearance of the liver parenchyma (4). Clinically, it manifests with a frequently asymptomatic rise in alkaline phosphatase (ALP), with or without changes in aspartate transaminase (AST) or alanine transaminase (ALT). These changes may be present for years, followed by jaundice, portal hypertension, and varices *via* nodular compression of sinusoids, portal, and central vasculature (7, 8). Given the association of NRH with portal hypertension, it follows that CVID patients with NRH also demonstrate increased morbidity and mortality as compared to the general CVID population (2, 9, 10). Currently, there are no Food and Drug Administration (FDA)-approved treatment modalities for NRH in CVID patients, although biologics are being tried for the treatment of various autoinflammatory end-organ complications in primary immunodeficiencies, making early diagnosis and potential early intervention the goal in CVID patient management (10–13).

The gold standard for diagnosis of NRH is a liver biopsy. In patients with CVID, NRH is characterized histologically by a nodular pattern of alternating areas of hepatic plate expansion and atrophy. NRH-like changes are often accompanied by other histologic features of CVID such as a sinusoidal lymphocytic infiltrate, mild portal and lobular inflammation, and variably prominent sinusoidal fibrosis (6). NRH-like changes may be present on liver biopsy even when clinical manifestations are subtle (such as mildly elevated liver enzymes), making improved diagnostic modalities essential. Furthermore, delays in liver biopsy procedures are common in patients with CVID due to 1) the aforementioned subtle presentation of NRH and 2) avoidance of high-risk procedures in immunodeficient patients (7). Therefore, patients frequently present with advanced disease with 19%–50% demonstrating manifestations of portal hypertension, such as gastroesophageal varices or cirrhosis, at the time of diagnosis (5, 8, 10).

Imaging modalities investigated to date for the diagnosis of NRH among CVID patients include CT, MRI, and ultrasound (US) (14, 15). Specific advantages of US imaging include being fast, low-cost, non-invasive, and in line with the goal to reduce repeat exposure to radiation in CVID patients, who are already at higher risk for both hematologic and solid-organ malignancies as compared to the general population (16). Traditionally, US with Doppler has been employed in patients with liver disease; however, non-specific findings limit its utility, and the detection of portal hypertension and splenomegaly is a late-stage finding. Vibration-controlled transient elastography (TE) or FibroScan^®^ uses transducer-induced vibrations to create shear waves that move throughout the liver parenchyma. Calculations of the speed of these shear waves can estimate the degree of liver stiffness (17). This approach has been well validated in chronic viral hepatitis to detect cirrhosis and stratify risk for portal hypertension and varices (18, 19). Additional data have demonstrated significant clinical utility in the diagnosis of non-alcoholic fatty liver disease (NAFLD) (20, 21). TE has been studied, generally, in NRH with variable results (14). More recently, this modality has demonstrated promise in the non-invasive detection of liver disease in patients with CVID, particularly those with lymphoproliferative and enteropathy phenotypes (22).

Given the prevalence of NRH in CVID patients with liver disease, we hypothesized that TE may be a non-invasive imaging tool that has diagnostic utility. In this study, we performed TE among CVID patients and analyzed liver stiffness by kPa, comparing groups with and without biopsy-proven NRH. In addition, we analyzed pathologic and clinical features, such as the severity of portal hypertension, which correlated with the degree of liver stiffness by TE among CVID patients. Finally, we created and analyzed receiver operating characteristic (ROC) curves to define a liver stiffness cutoff by TE with diagnostic utility for NRH among CVID patients.

## Methods

### Participants

This study was performed at Mass General Brigham under an Institutional Review Board-approved protocol (#2011P000940).

Participants were recruited from Massachusetts General Hospital Immunology and Gastroenterology clinics from January 1, 2018, to March 14, 2022. CVID was diagnosed using International Consensus Document (ICON) criteria (1). NAFLD was diagnosed using the American Association for the Study of Liver Diseases (AASLD) guidelines (23). In a CVID cohort followed up longitudinally at our single-center institution, a consecutive sample of CVID patients with abnormal liver biochemistries (AST > 40 U/L, ALT > 55 U/L, or ALP > 100 U/L) and biopsy-proven NRH were offered TE (N = 12). CVID patients without NRH (defined as no abnormal liver biochemistries (N = 4) or abnormal liver biochemistries biopsied negative for NRH (N = 2)) were offered TE. At the time of initial enrollment, CVID participants had not received active immunosuppressive therapy for an end-organ lymphoinfiltrative disease related to CVID. One CVID patient with NRH did receive a single dose of abatacept therapy (500 mg intravenously) 1 month prior to the time of TE. One CVID patient with NRH was on chronic mycophenolate mofetil (1,000 mg twice daily) for neuromyelitis optica. The remainder of the CVID participants received no B-cell or T-cell suppressive therapy in the 6 months prior to the time of TE. A total of N = 527 patients with NAFLD were included in a comparator cohort. Exclusion criteria were any evidence of hepatitis virus infection (defined as a positive viral load by PCR), alcoholic cirrhosis, and/or moderate ascites at the time of imaging. Additionally, in the NAFLD cohort, previously diagnosed autoimmune hepatitis was an exclusion criterion.

### Measurement of Liver Stiffness

TE using FibroScan^®^ was performed by a trained ultrasonographer who completed 10 serial measurements with a median score reported. The raw median score in kPa was used for all subsequent analyses. In CVID patients, a ROC curve was generated to define the most accurate diagnostic kPa cutoff for NRH. A cutoff of ≥7.5 kPa was also assessed given its clinical significance in determining elevated liver stiffness among patients diagnosed with NAFLD (24). Controlled attenuation parameter (CAP) values (dB/m) were evaluated to quantify the relationship between liver stiffness and hepatic steatosis in those patients (N = 10) who had this value calculated.

### Liver Histopathology

Liver biopsy slides directly available at our institution were included in the histopathologic analysis (N = 7) and were reviewed by a gastrointestinal (GI) pathologist blinded to the TE data and the initial pathology report. H&E and trichrome-stained slides were reviewed for each case. Histopathology was deemed to be generally consistent, or not, with NRH and additionally was classified based on the following characteristics: the number of nodules identified (N), diameter of the largest nodule (mm), nodule length (mm), nodule density (nodules/10 mm of core length), thickest hepatic plate (N cell layers), sinusoidal dilatation (±), sinusoidal lymphocytosis (±), centrilobular fibrosis, and/or portal fibrosis. Fibrosis was graded as absent, focal, diffuse, bridging, or cirrhosis.

### Collection and Definition of Clinical and Laboratory Data

Participants’ clinical data were extracted from the electronic medical record. For all CVID participants, this included a review of complete blood count (CBC) with differential and liver biochemistries (AST, ALT, ALP, gamma-glutamyl transferase (γ-GGT), total bilirubin, albumin, coagulation factors (prothrombin/partial thromboplastin time), and ammonia). Peak ALP was also recorded, included in the absence of other acute causes of elevation (e.g., infection and acute clinical decompensation). Patient immunophenotype was reviewed including immunoglobulin levels, peripheral flow cytometry including T-cell, B-cell, NK-cell, class-switched memory B-cell, and naïve/memory T-cell counts (absolute and relative percentages in peripheral blood) closest to the time of TE measurement. T-cell proliferation to mitogens, antigens, and anti-CD3 were also reviewed. Clinical parameters of liver disease were reviewed by the electronic medical recorded diagnosis, as follows: the presence of clinical portal hypertension, varices (grade 1–3), ascites (trace only), and/or peripheral edema. In individuals with transjugular liver biopsies, hepatic venous pressure gradient (HVPG) measurements were evaluated (N = 8). HVPG >5 and >10 mmHg were used to define any portal hypertension and clinically significant portal hypertension, respectively (25). In CVID participants with abdominal CT imaging available (N = 13), spleen size was measured using the largest anterior–posterior diameter on axial imaging, with those ≥12 cm considered enlarged (26). Four CVID participants had prior splenectomy and were excluded from this portion of the analysis. Prior infections specifically reviewed included hepatitis viruses (hepatitis A, B, and C), Epstein–Barr virus (EBV), cytomegalovirus (CMV), and Giardia. Autoinflammatory CVID complications were defined as the presence of autoimmune enteropathy, autoimmune cytopenias, chronic (>6 months) lymphadenopathy, and/or granulomatous–lymphocytic interstitial lung disease (GLILD). Route of replacement immunoglobulin and dose by body weight were also recorded.

CVID was subcategorized as complicated or uncomplicated. Complicated CVID was defined by the presence of any autoinflammatory clinical feature (described above) or the presence of a combined deficiency immunophenotype (class-switched memory B cells <2% of total CD19+ B cells and naïve CD4+CD45RA+ T cells <20% of total CD4+ T cells).

For all data recorded above, the measurement closest to the time of the TE was recorded. The median time from TE to liver biopsy was 359 days (Q1–Q3, 163–890 days), to immunoglobulin level was 162 days (72–408 days), to flow cytometry was 185 days (78–675 days), to liver biochemistry was 39 days (15–147 days), and to CBC was 49 days (28–112 days).

### Statistical Analysis

Data are represented as means ± SD, median (Q1–Q3), or proportions unless otherwise noted. The relationship between liver stiffness with immunologic and clinical variables was measured using one-way ANOVA with Tukey’s post-hoc correction and simple linear regression. A Mood’s median test compared median liver stiffness measurements. The chi-square test and logistic regression were used to compare categorical data. To account for the small sample size, log transformation was applied to continuous variables and Fisher’s exact test to categorical variables when estimating p-values. If linear regression assumptions were not met after transformation, Spearman’s correlation was performed. Figures were created utilizing GraphPad Prism 9.3.1 (GraphPad Software, San Diego, CA, USA) or BioRender.com. Statistical analyses were completed with SAS 9.4 (SAS Institute, Cary, NC, USA); a two-tailed p-value of <0.05 was considered significant.

## Results

### Characteristics of Patients Who Underwent Transient Elastography

TE was performed at our single-center institute on 12 CVID patients with NRH, 6 CVID patients without NRH, and 527 non-CVID patients with NAFLD. The median age of all participants was 55 (Q1–Q3, 46–64) years, and 55.7% were female. There were no significant differences in age, race, and sex in the subgroups of CVID with NRH, CVID without NRH, and non-CVID with NAFLD. Within CVID subgroups, there was no significant difference between age at diagnosis and time from diagnosis to completion of liver stiffness measurement (**Supplementary Table 1**).

Immunophenotypes of CVID patients with and without NRH who underwent TE were evaluated (**Supplementary Table 2**). CVID patients with NRH had lower naïve CD4+CD45RA+ T cells, by both absolute count and percentage (84 vs. 276, cells/μl, 18% vs. 43%; p = 0.04). There were no significant differences in the remainder of lymphocyte subsets, immunoglobulin levels, or T-cell functional studies analyzed, although there was a trend towards lower class-switched memory B cells in CVID patients with NRH (1.4% vs. 3.1%; p = 0.11).

Clinical complications and immunoglobulin treatment differences among CVID patients with and without NRH who underwent TE were compared (**Supplementary Table 3**). A larger proportion of CVID patients with NRH had autoinflammatory complications compared to CVID patients without NRH (83% vs. 17%; p = 0.01). Specifically, CVID patients with NRH more often had additional diagnoses of GLILD and lymphadenopathy as compared to CVID patients without NRH (67% vs. 0%; p = 0.01, 58% vs. 0%; p = 0.04, respectively). In addition, there was a trend toward a higher dose per body weight of replacement immunoglobulin in those with NRH, compared to those without (mean 699 vs. 485, mg/kg/month; p = 0.05). Consistent with these immunophenotypic and clinical data, all (N = 12) CVID patients with NRH met the classification for complicated CVID, while only 1 of 6 CVID patients without NRH similarly met the classification for complicated CVID (**Supplementary Table 1**).

Finally, we compared markers of liver disease in CVID patients with and without NRH who underwent TE (**Supplementary Table 4**). We identified higher levels of AST (54 vs. 26, U/L; p < 0.01), ALP proximal to TE (250 vs. 100, U/L; p = 0.03), peak ALP (314 vs. 114, U/L; p = 0.02), albumin (4.3 vs. 3.9, g/dl; p = 0.02), and total bilirubin (0.75 vs. 0.35, mg/dl; p = 0.01) in CVID patients with diagnosed NRH compared to those without NRH. Several markers of portal hypertension additionally were associated with diagnosed NRH, including increased splenic longitudinal diameter (16.6 vs. 11.2, cm; p = 0.02), presence of any grade varices (67% vs. 0%; p = 0.04), and clinical portal hypertension diagnosed in the electronic medical record (83% vs. 0%; p < 0.01).

### Patients With Common Variable Immunodeficiency and Nodular Regenerative Hyperplasia Have Elevated Liver Stiffness by Transient Elastography

**Figure 1:**
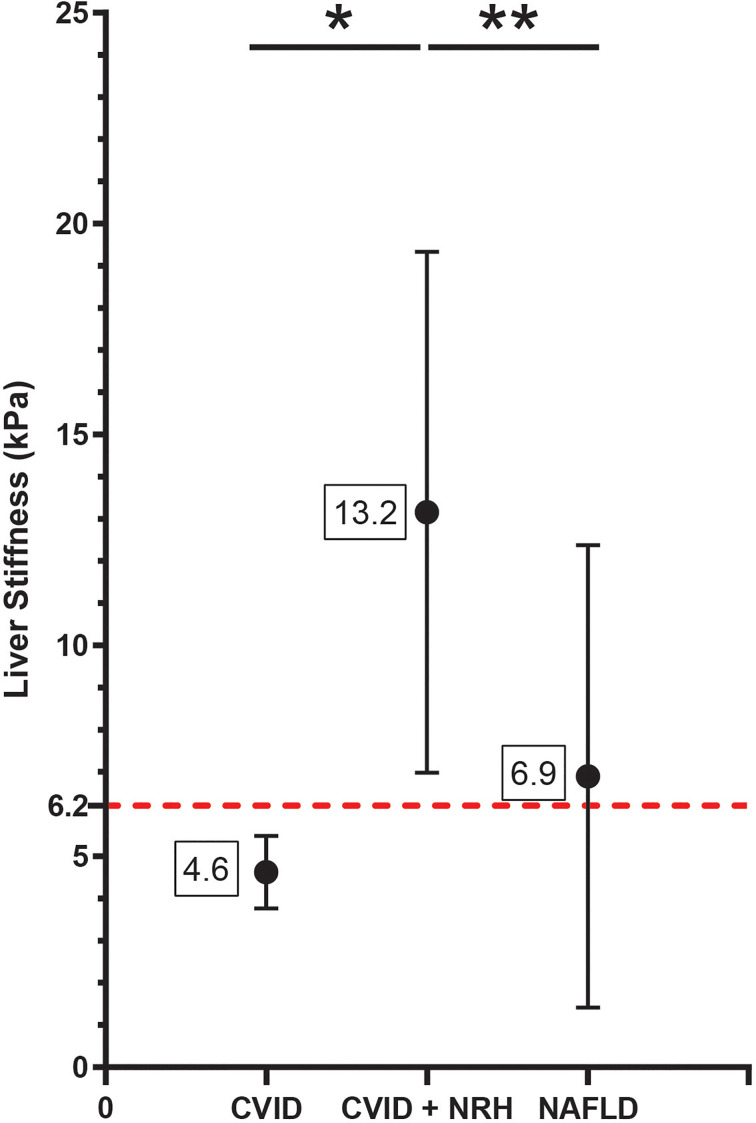
Liver stiffness by transient elastography is significantly elevated in CVID patients with NRH. Liver stiffness measurements (kPa) by transient elastography shown as mean (± SD) in CVID patients with biopsy-proven NRH (CVID + NRH, N = 12), CVID patients without NRH (CVID, N = 6), and non-CVID patients with non-alcoholic fatty liver disease (NAFLD, N = 527). Significance by one-way ANOVA with Tukey’s post-hoc correction; *, p = 0.01; **, p < 0.01. Red dotted line indicates a diagnostic cutoff value for liver stiffness of 6.2 kPa (defined using the ROC curve in **Figure 4**). CVID, common variable immunodeficiency; NRH, nodular regenerative hyperplasia; ROC, receiver operating characteristic.

Next, we compared measures of liver stiffness by TE among CVID patients with and without NRH. As an additional disease comparator, we included a cohort of patients followed up at our center for NAFLD, a disease where TE is already used in routine clinical diagnosis. We identified a significantly higher measure of liver stiffness in CVID patients with NRH compared to CVID patients without NRH and non-CVID patients with NAFLD (**Figure 1**). CVID participants with diagnosed NRH had a mean liver stiffness of 13.2 (± 6.2) kPa and a median liver stiffness of 11.9 (8.4–18.1) kPa. In contrast, CVID participants without NRH had a mean liver stiffness of 4.6 (± 0.9) kPa and median liver stiffness of 4.6 (4.1–5.3) kPa (p = 0.01, CVID with NRH vs. without NRH). Finally, non-CVID NAFLD patients had a mean liver stiffness of 6.9 ( ± 5.5) kPa and median liver stiffness of 5.5 (4.3–7.1) kPa (p < 0.01, CVID with NRH vs. NAFLD). Liver stiffness was positively correlated with CAP measurements, although this relationship was not significant (r = 0.53; p = 0.12) (**Supplementary Figure 1**).

### Liver Stiffness by Transient Elastography Does Not Correlate With Any Specific Histopathologic Feature of Nodular Regenerative Hyperplasia Among Common Variable Immunodeficiency Patients

**Figure 2:**
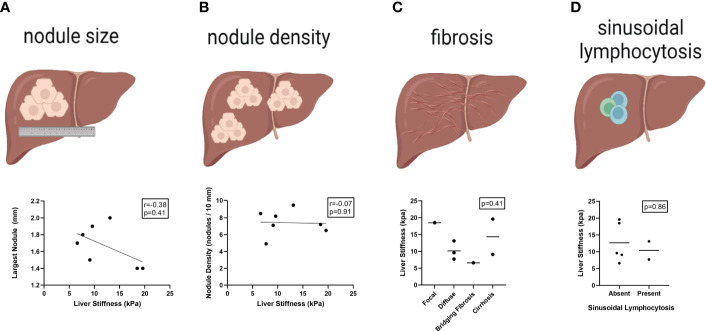
Liver stiffness by transient elastography does not correlate with specific histopathologic features of NRH in CVID patients. Liver stiffness measurements (kPa) by transient elastography compared across histopathologic features of NRH in CVID patients with available liver biopsy (N = 7), including size of largest nodule **(A)**, nodule density **(B)**, centrilobular fibrosis **(C)**, and sinusoidal lymphocytosis **(D)**. Significance by one-way ANOVA with Tukey’s post-hoc correction or Spearman’s correlation (r) with significance (p) shown. Line of best fit **(A, B)** and mean value **(C, D)** are shown. NRH, nodular regenerative hyperplasia; CVID, common variable immunodeficiency.

NRH has been associated with a diverse spectrum of histopathologic findings (27, 28). A detailed review of liver histopathology was performed to determine which specific features may be driving the observed increased measure of liver stiffness in CVID patients with NRH. Using blinded scoring, we did not observe any appreciable association between liver stiffness measurements and nodule size, nodule density, fibrosis, or sinusoidal lymphocytosis (**Figure 2**). A composite score of these histopathologic variables was created to minimize redundancy and maximize concordance during interpretation. Again, there was no significant association between composite scoring and liver stiffness measurements by TE.

### Liver Stiffness by Transient Elastography Significantly Correlates With Portal Hypertension Among Common Variable Immunodeficiency Patients

As we observed no association between histopathologic features and liver stiffness by TE in CVID patients with NRH, we alternatively evaluated whether the observed increased liver stiffness in this population was driven by the severity of physiologic portal hypertension.

**Figure 3:**
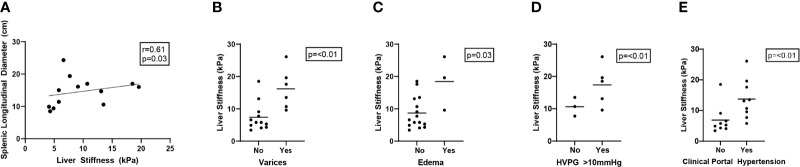
Liver stiffness by transient elastography correlates with clinical parameters of portal hypertension in CVID patients. Liver stiffness measurements (kPa) by transient elastography compared across clinical parameters of portal hypertension in CVID patients, including splenic longitudinal diameter (**A**, N = 13 scored), presence of varices (grade 1–3) (**B**, N = 18 scored), presence of peripheral edema (**C**, N = 18 scored), elevated (>10 mmHg) hepatic venous pressure gradient (HVPG) (**D**, N = 8 scored), and clinically diagnosed portal hypertension in the electronic medical record based on any combination of these data (**E**, N = 18 scored). Significance by one-way ANOVA with Tukey’s post-hoc correction or Spearman’s correlation (r) with significance (p) shown. Line of best fit **(A)** and mean value **(B–E)** are shown. CVID, common variable immunodeficiency.

Among all CVID participants, patients with clinical evidence of portal hypertension were found to have significantly higher liver stiffness measurements by TE. Specifically, there was a significant association between liver stiffness and splenic longitudinal diameter (r = 0.61; p = 0.03), presence of grade 1–3 varices (mean kPa 16.5 vs. 6.4, median kPa 17.6 vs. 5.8; p < 0.01), and presence of peripheral edema (mean kPa 18.4 vs. 8.7, median kPa 19.6 vs. 6.6; p = 0.03, p=0.07, respectively). Additionally, liver stiffness was higher among CVID patients with diagnosed portal hypertension, either clinically (mean kPa 14.2 vs. 5.4, median kPa 13.3 vs. 5.1; p < 0.01) or by increased HVPG (mean kPa 15 vs. 7.7, median kPa 13.1 vs. 5.5; p < 0.01) (**Figure 3**, **Table 1**). In contrast, CAP measurements by TE did not significantly differ across clinical parameters of portal hypertension (**Supplementary Table 5**).

**Table 1:** Liver stiffness by transient elastography correlates with clinical markers of portal hypertension.

	kPa (mean)	p-Value	kPa (median)	p-Value
**Clinical parameters of portal hypertension**				
Splenic longitudinal diameter				
≥12 cm	12.1		13.1	
<12 cm	5.8	0.04	5.3	0.11
Splenectomy				
Yes	14.5		12.1	
No	9.1	0.13	6.2	0.27
Hepatic venous pressure gradient				
>5 mmHg	15		13.1	
≤5 mmHg	7.7	<0.01	5.5	<0.01
>10 mmHg	17.4		18.5	
≤10 mmHg	7.9	<0.01	5.5	0.02
Varices				
Yes	16.5		17.6	
No	6.4	<0.01	5.8	<0.01
Ascites				
Yes	16.4		13.5	
No	9.1	0.08	6.6	0.07
Edema				
Yes	18.4		19.6	
No	8.7	0.03	6.6	0.07
Clinical portal hypertension				
Yes	14.2		13.3	
No	5.4	<0.01	5.1	<0.01
**Autoinflammatory comorbidity**				
Yes	11.3		9.6	
No	8.8	0.22	5.3	0.16
GLILD				
Yes	11.1		9.4	
No	9.7	0.41	5.5	0.36
Lymphadenopathy				
Yes	12.4		10.7	
No	9.0	0.17	5.8	0.16
Enteropathy				
Yes	13.1		10.2	
No	9.5	0.36	7.2	0.27
Cytopenia				
Yes	10.3		10.4	
No	10.3	0.78	7.9	1
**Gastrointestinal infection**				
Yes	12.6		9.2	
No	9.8	0.47	7.9	1
**Replacement immunoglobulin**				
SCIG	9.2		5.8	
IVIG	11	0.54	9.1	0.64

CVID, common variable immunodeficiency; NRH, nodular regenerative hyperplasia; kPa, kilopascal; GLILD, granulomatous lymphocytic interstitial lung disease; SCIG, subcutaneous immunoglobulin; IVIG, intravenous immunoglobulin.

Given the higher prevalence of complicated CVID in patients with versus without NRH who underwent TE, we evaluated if other clinical parameters correlated with the measure of liver stiffness in this patient demographic. The presence of CVID-related autoinflammatory disease was not associated with higher liver stiffness by TE. Specifically, there was no difference in a mean or median liver stiffness between CVID patients with GLILD, lymphadenopathy, enteropathy, or cytopenias. Additionally, liver stiffness values did not differ based on the history of GI infection or type of replacement immunoglobulin received (**Table 1**).

### Liver Stiffness and Alkaline Phosphatase Have Utility in the Diagnosis of Nodular Regenerative Hyperplasia Among Common Variable Immunodeficiency Patients

**Figure 4:**
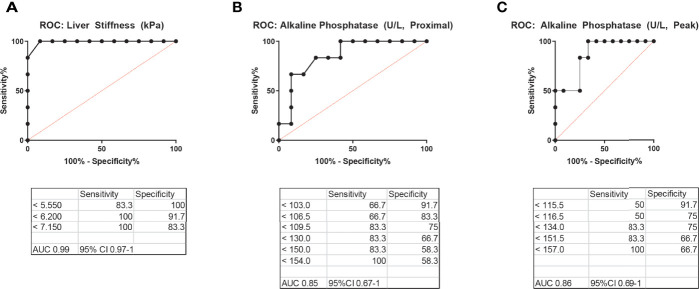
Receiver operating characteristic (ROC) curves for the diagnosis of NRH in CVID patients. ROC curves for **(A)** liver stiffness by transient elastography, **(B)** alkaline phosphatase (ALP) level in peripheral blood most proximal to the time of transient elastography, and **(C)** peak ALP level in peripheral blood, excluding acute illness, to diagnose NRH in patients with CVID. AUC, area under the curve; NRH, nodular regenerative hyperplasia; CVID, common variable immunodeficiency.

Previously, an ALP level in peripheral blood >1.5 times the upper limit of normal was suggested as a useful marker in the diagnostic workup of NRH for CVID patients, specifically to prompt further liver biopsy (7). We sought to comparatively analyze the diagnostic utility of liver stiffness by TE versus ALP in our cohort of CVID patients with and without NRH. We created ROC curves for liver stiffness (kPa) and ALP (proximal to the time of TE and peak level) (**Figure 4**). Liver stiffness had robust ROC curve parameters, with an area under the curve (AUC) of 0.99 (95%CI 0.97–1) and a cutoff of 6.2 kPa demonstrating excellent performance by sensitivity and specificity for prediction of NRH in CVID. ROC curves for proximal ALP and peak ALP were both adequate diagnostic tests, albeit with lower AUCs of 0.85 (95%CI 0.67–1) and 0.86 (95%CI 0.69–1), respectively. An ALP of 154 U/L (proximal) and 157 U/L (peak) had a sensitivity of 100% for the detection of NRH in CVID, although lacked specificity (58% and 67%, respectively).

**Figure 5:**
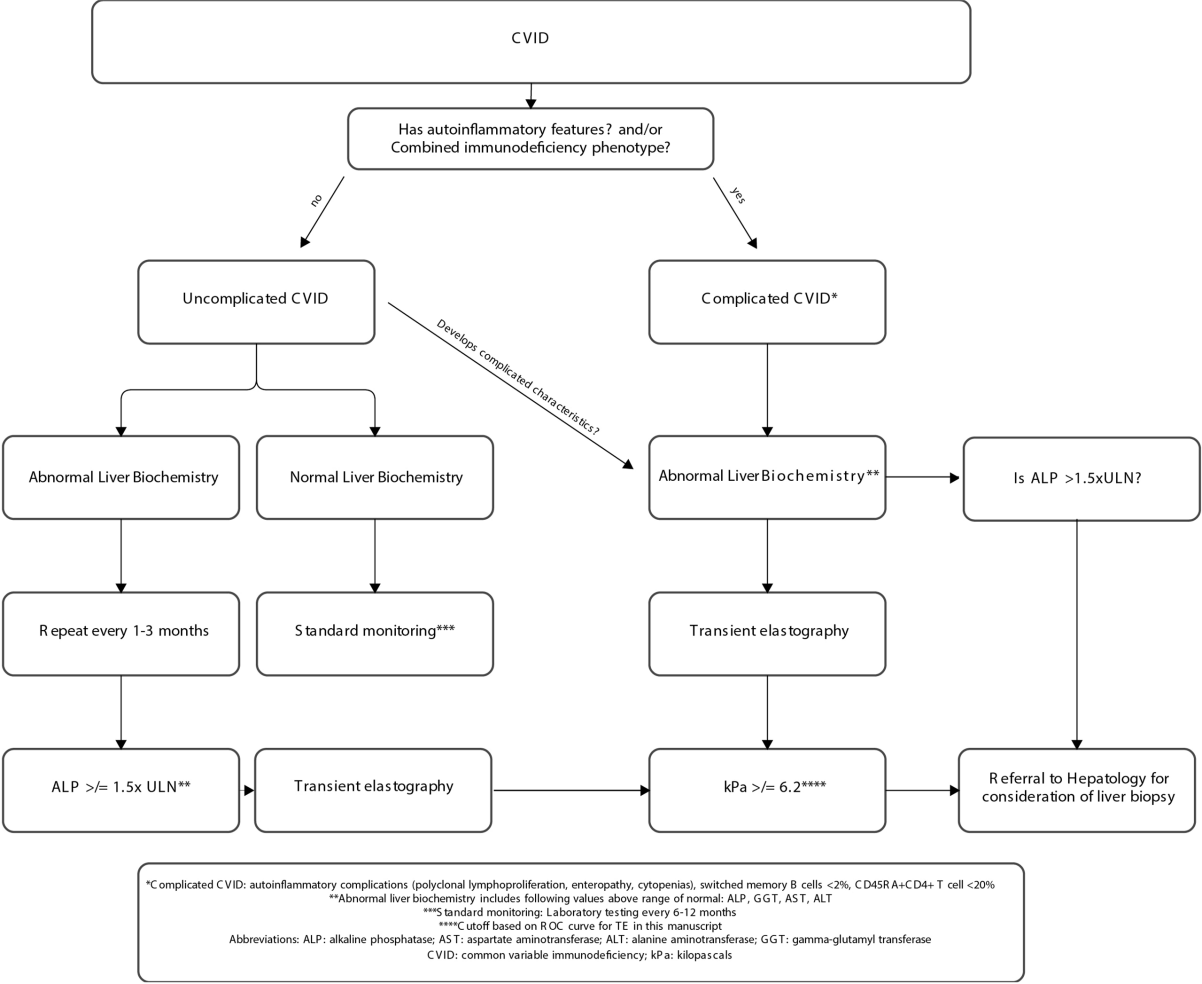
Clinical algorithm for early detection of nodular regenerative hyperplasia in individuals with CVID. CVID, common variable immunodeficiency; ULN, upper limit normal.

Finally, we analyzed peripheral blood immunophenotypes and liver biochemistries, comparing the liver stiffness cutoffs of 6.2 kPa (defined by our ROC above) and 7.5 kPa [a threshold for increased liver stiffness previously used in NAFLD (24)] (**Tables 2**, **3**). CVID patients with ≥6.2 kPa had significantly lower mean absolute CD4+CD45RA+ naïve T cells (82 vs. 241 cells/μl; p = 0.04), higher AST levels (53 vs. 33 U/L; p = 0.04), and higher ALP levels (proximal, 259 vs. 107 U/L; p = 0.04; peak, 328 vs. 121 U/L; p = 0.01). The only significant comparisons using a cutoff of ≥7.5 kPa were albumin, which was lower (3.8 vs. 4.3 mg/dl) in the high kPa group, and peak ALP, which was higher (335 vs. 138 U/L) in the high kPa group. Otherwise, there were no significant differences in immunoglobulin levels, lymphocyte subsets, or liver biochemistries between the two groups. Together, these data suggest that a liver stiffness measurement by TE of ≥6.2 kPa is an accurate diagnostic cutoff for NRH in patients with CVID. We developed a clinical algorithm for the early detection of NRH in patients with CVID utilizing this diagnostic cutoff (**Figure 5**).

**Table 2:** Relationship between peripheral blood immunophenotypes and liver stiffness cutoffs for NRH in CVID patients.

	kPa ≥ 6.2	kPa < 6.2	p-Value	kPa ≥ 7.5	kPa < 7.5	p-Value
**Immunoglobulins [mean (mg/dl)]**						
IgG	1038	943	0.63	1029	967	0.50
IgA	39	85	0.19	42	76	0.29
IgM	315	43	0.17	323	66	0.35
**Flow cytometry [mean (cells/μl), %]**						
CD3+	1319, 72	1041, 70	0.71	1401, 73	973, 69	0.99
CD4+	857, 46	770, 51	0.47	917, 47	708, 47	0.80
CD8+	407, 21	239, 17	0.87	428, 21	232, 17	0.77
CD3−CD16+56+	188, 11	181, 12	0.42	175, 8	199, 15	0.21
CD4+CD45RA+	82, 17	241, 38	0.043	91, 19	201, 32	0.54
CD4+CD45RO+	530, 77	318, 55	0.88	560, 75	308, 62	0.78
CD8+CD45RA+	136, 54	167, 66	0.234	148, 59	148, 57	0.96
CD8+CD45RO+	92, 36	62, 27	0.84	87, 32	74, 34	0.77
CD19+	616, 15	223, 16	0.92	676, 16	197, 14	0.73
CD19+CD27+	17, 9	54, 21	0.14	18, 7	47, 23	0.18
CD19+CD27+IgM/IgD−	1.7, 1.5	6.5, 2.6	0.28	1.8, 0.8	5.7, 3.4	0.33

Significance by one-way ANOVA shown.

CVID, common variable immunodeficiency; NRH, nodular regenerative hyperplasia; kPa, kilopascal.

^*^T-cell function excluded as N = 6–12 missing for each variable.

**Table 3:** Relationship between liver biochemistries and liver stiffness cutoffs for NRH in CVID patients.

	kPa ≥ 6.2	kPa < 6.2	p-Value	kPa ≥ 7.5	kPa < 7.5	p-Value
Liver biochemistries^*^						
AST (U/L)	53	33	0.04	52	36	0.12
ALT (U/L)	43	30	0.32	44	30	0.31
ALP (U/L, proximal)	259	107	0.04	259	126	0.1
ALP (U/L, peak)	328	121	0.01	335	138	0.03
γ-GGT (U/L)^**^	151	518	0.18	293	168	0.66
Albumin (g/dl)	3.9	4.2	0.1	3.8	4.3	0.01
Total bilirubin (mg/dl)	0.73	0.44	0.08	0.76	0.43	0.05
PT/PTT						
Abnormal (%)	9	0	1	10	0	1

Significance by one-way ANOVA shown.

CVID, common variable immunodeficiency; NRH, nodular regenerative hyperplasia; kPa, kilopascal.

^*^Ammonia level not collected on any participants.

^**^γ-GGT, N = 7 participants with data.

## Discussion

This study demonstrates the utility of TE in diagnosing NRH among patients with CVID. While prior investigations have analyzed the relationship between TE and liver disease in CVID, this is the first study to our knowledge to do so in those with biopsy-confirmed NRH.

It has been established that liver stiffness measurements are elevated in CVID-related liver disease. Crescenzi et al. previously investigated TE in CVID patients with liver disease demonstrating a mean liver stiffness of 7.5 kPa, with 75% of participants meeting the definition of complicated CVID used in this study. In the absence of enteropathy or polyclonal lymphoproliferation, the mean liver stiffness appeared to be below 6.2 kPa (22). Prior studies have also demonstrated an association of elevated liver stiffness with CVID phenotypes such as polyclonal lymphoproliferation (GLILD, persistent lymphadenopathy, and granuloma) and enteropathy, as well as markers of portal hypertension (e.g., splenic longitudinal diameter) (5, 7, 22). The most established predictor of NRH in CVID is an elevated ALP level, although any of the liver biochemistry tests may be abnormal. Ward et al. eloquently demonstrated that ALP levels in NRH can follow several different patterns, the most common being a steady increase over time, and this generally starts several years after CVID diagnosis (7). Importantly, 30% or more of patients with liver disease in CVID may have normal liver biochemistries (29). It is known that liver disease in CVID is a poor prognostic indicator and that NRH specifically is associated with portal hypertension complications such as hemoptysis and ascites (4, 5, 22).

In this study, we identified significantly elevated liver stiffness by TE in CVID patients with NRH compared to both CVID patients without NRH and non-CVID patients with NAFLD. Interestingly, we did not find a statistically significant association between histopathologic features of NRH on liver biopsy and liver stiffness by TE in our CVID patient demographic. This is consistent with other studies of NRH in non-CVID populations, where liver stiffness by TE has not been associated with specific histopathologic features (14). This lack of concordance between NRH histopathology on biopsy and liver stiffness by TE could be due to multiple factors including limited consensus criteria in the histopathologic definition of NRH or adequacy of biopsy samples (28). In contrast, we did demonstrate a significant association between liver stiffness by TE and several clinical measures of portal hypertension in this study. These data suggest that TE has specific utility in identifying progression to portal hypertension among CVID patients with NRH, which is of great clinical importance given the concomitant increase in CVID patient morbidity and mortality associated with this clinical progression (4, 22).

There are several limitations and sources of bias to consider in this study. While all eligible CVID participants with NRH were recruited, not all of those without NRH participated in the study. This may introduce selection bias in that CVID patients without NRH who consented to be included may be different from those who did not. This study is also cross-sectional in nature, and liver stiffness levels were not followed up over time, limiting causal inference. Furthermore, the study population was relatively small with recruitment from an academic tertiary care center, limiting statistical power and adding to potential selection bias or increasing type I error. Given the rarity of CVID, though, this is not unexpected and is a difficult limitation to overcome. It is important to note that four participants had splenectomy before this study, and thus, splenic diameter measurements were unavailable. As those with prior splenectomy likely had severe disease, censoring these patients in our analysis would have biased results towards the null, minimizing an association. While we did incorporate a comparison between liver stiffness by TE in those with CVID and NAFLD, mean liver stiffness measurements in NAFLD participants were below the established threshold of clinically significant fibrosis. We were unable to correlate liver stiffness measurements in those with NAFLD to the extent of fibrosis on biopsy given our data set. The influence of steatosis and other pathologic processes unrelated to NRH on liver stiffness measurements is difficult to measure and must be acknowledged as well. We attempted to correlate CAP as a surrogate of hepatic steatosis to liver stiffness and markers of portal hypertension, and while there did seem to be a positive correlation, this was not statistically significant, which we believe was due to the small sample size. Finally, most participants with NRH in this study had severe disease, with evident portal hypertension. It is therefore difficult to make conclusions related to the utility of TE as a predictor of early NRH. Future studies investigating the impact of early NRH diagnosis and the impact of underlying complicated CVID features in those without liver disease will be of great importance.

The current standard of care in patients with CVID and NRH suffers from diagnostic delays and treatments that carry high morbidity and mortality (i.e., transplant) (10, 11). TE is a potentially helpful tool for the diagnosis and monitoring of liver disease through the progression of disease and response to treatment in NRH. Based on the findings in this study, we propose an algorithm that utilizes CVID immunophenotype, liver biochemistries, and liver stiffness by TE to stratify NRH risk among CVID patients. Specifically, for patients with features consistent with complicated CVID, we proposed that any abnormal liver biochemistry measurement should trigger the measurement of liver stiffness by TE. Furthermore, the patient should be referred to hepatology for consideration of liver biopsy if either of the following criteria are met: the measure of liver stiffness by TE is at or above 6.2 kPa or the ALP level (peak) is >1.5× the upper limit of normal (**Figure 5**). Future prospective studies are needed and should incorporate individuals with elevated ALP and uncomplicated CVID. Recognition of early disease, as well as confirmatory biopsy, has the potential to improve our ability to recognize and/or prognosticate regarding the timing of complications, such as portal hypertension, in CVID patients with NRH. As immunomodulatory therapies become increasingly available for the treatment of immune dysregulation in CVID, there is an opportunity for improved treatment and management as our understanding of the disease process that leads to elevated liver stiffness in NRH improves.

## Data Availability Statement

The raw data supporting the conclusions of this article will be made available by the authors, without undue reservation.

## Ethics Statement

This study was performed at Mass General Brigham under an Institutional Review Board-approved protocol (#2011P000940). The patients/participants provided their written informed consent to participate in this study.

## Author Contributions

DD wrote the manuscript and performed the chart review and statistical analyses on the cohort. JS, PB, and KC provided patients from the gastroenterology clinic, as well as clinical expertise. RC reviewed liver biopsy histopathology and provided expertise in the creation of biopsy scoring for NRH. NY performed the chart review. JF conceived the project and supervised all aspects of the work. SB and JF provided expertise in immunology. All authors reviewed the manuscript, approved the final manuscript as submitted, and agree to be accountable for all aspects of the work.

## Funding

SB is supported by the National Institute of Allergy and Infectious Diseases of the National Institutes of Health under Award Number K23AI163350.

## Author Disclaimer

The content is solely the responsibility of the authors and does not necessarily represent the official views of the National Institutes of Health.

## Conflict of Interest

JF holds investigator-initiated grants from Bristol Myers Squibb and Pfizer with no direct relation to the work presented.

The remaining authors declare that the research was conducted in the absence of any commercial or financial relationships that could be construed as a potential conflict of interest.

## Publisher’s Note

All claims expressed in this article are solely those of the authors and do not necessarily represent those of their affiliated organizations, or those of the publisher, the editors and the reviewers. Any product that may be evaluated in this article, or claim that may be made by its manufacturer, is not guaranteed or endorsed by the publisher.

## Abbreviations

CVID, common variable immunodeficiency; NRH, nodular regenerative hyperplasia; NAFLD, non-alcoholic fatty liver disease; kPa, kilopascal
