# Supplementary material for: Liver Stiffness by Transient Elastography Correlates With Degree of Portal Hypertension in Common Variable Immunodeficiency Patients With Nodular Regenerative Hyperplasia
Source: Front Immunol. 2022 May 6;13:864550. doi: 10.3389/fimmu.2022.864550 (PMC9121126; doi:10.3389/fimmu.2022.864550)
Supplement: Supplementary file 1 [file DataSheet_1.docx]

**Supplemental:**

**Supplemental Table 1.** **Characteristics of patients who underwent transient elastography.**

|  | **CVID with NRH (N=12)** | **CVID without NRH (N=6)** | **NAFLD**  **(N=527)** | **p-value** |
| --- | --- | --- | --- | --- |
| Age (years, median)  (Q1-Q3) | 45  (34.5-64.5) | 60  (48-65) | 55  (46-64) | 0.60 |
| Sex  Female (%) | 50 | 100 | 55.4 | 0.07 |
| Race  Non-Hispanic White (%) | 100 | 100 | 72.9 | 0.87 |
| Age at CVID diagnosis (years, median)  (Q1-Q3) | 41  (20-64) | 56.6  (47-58) | - | 0.052 |
| Time from CVID diagnosis to liver stiffness measurement (years, median)  (Q1-Q3) | 4.5  (2-13) | 2.5  (2-4) | - | 1 |
| kPa  Mean  Median  >/=6.2 (%)  >/=7.5 (%) | 13.2  11.9  92  83 | 4.6  4.6  0  0 | 6.9  5.5  37  22 | <0.01  <0.01  <0.01  <0.01 |
| Diagnosis (%)  Complicated CVID | 100 | 16.7 | - | <0.01 |
| T /B cell suppression (past 6 months, %)  Yes | 17 | 0 | - | 1 |

CVID: common variable immunodeficiency; NRH: nodular regenerative hyperplasia; NAFLD: nonalcoholic fatty liver disease; kPa: kilopascals. Significance by chi-squared with Fisher’s exact test shown.

**Supplemental Table 2. Comparison of peripheral blood immunophenotypes in CVID patients, with and without NRH, who underwent transient elastography.**

|  | **CVID with**  **NRH (N=12)** | **CVID without**  **NRH (N=6)** | **p-value** |
| --- | --- | --- | --- |
| **Hematology (mean)**  WBC (K/uL)  ANC (cells/uL)  ALC (cells/uL)  PLT (K/uL) | 6.7  3300  1683  224 | 7.0  4678  1618  222 | 0.48  0.19  0.55  0.34 |
| **Immunoglobulins (mean (mg/dL))**  IgG  IgA  IgM  Missing (N) | 1028  36  289  0 | 947  98  50  0 | 0.66  0.07  0.47 |
| **Flow cytometry (mean (cells/uL), %)**  CD3+  CD4+  CD8+  CD3-CD16+CD56+  CD4+CD45RA+  CD4+CD45RO+  CD8+CD45RA+  CD8+CD45RO+  CD19+  CD19+CD27+  CD19+CD27+IgM/IgD-  Missing (N)** | 1294, 72  831, 45  406, 22  181, 11  84, 18  520, 77  151, 60  90, 34  588, 15  28, 13  1.6, 1.4  1 | 1045, 70  809, 45  210, 15  194, 11  276, 43  292, 49  132, 64  58, 28  213, 15  38, 16  7.8, 3.1  2 | 0.75  0.42  0.61  0.36  0.04  0.74  0.76  0.70  0.90  0.49  0.11 |
| **T cell Function (% abnormal)**  Anti-CD3  PHA  PWN  Candida  Tetanus  Missing (N) | 0  11  0  8  75  (3-8) | 0  0  0  0  33  (3-4) | 1  1  1  1  1 |

p-value absolute # by one-way ANOVA; **Missing flow cytometry values refer to memory B and naïve/memory T cell panels; N=0 missing for T, B, NK cell enumeration. Significance by one-way ANOVA shown. CVID: common variable immunodeficiency; NRH: nodular regenerative hyperplasia; WBC: white blood cell count; ANC: absolute neutrophil count; ALC: absolute lymphocyte count; PLT: platelet count; PHA: phytohemagglutinin; PWN: pokeweed mitogen.

**Supplemental Table 3. Comparison of clinical features in CVID patients, with and without NRH, who underwent transient elastography.**

|  | **CVID with**  **NRH (N=12)** | **CVID without NRH (N=6)** | **p-value** |
| --- | --- | --- | --- |
| **Autoinflammatory Co-morbidity (yes, %)**  Any  GLILD  Lymphadenopathy  AIE  AI Cytopenia | 83  67  58  33  58 | 17  0  0  0  17 | 0.01  0.01  0.04  0.26  0.6 |
| **GI Infectious History (yes, %)**  Hepatitis (HAV, HBV, HCV)  EBV/CMV  Giardia | 0  33  0 | 0  0  0 | 0.25 |
| **IVIG/SCIG (yes, %)**  Yes  SCIG  Immunoglobulin dose (mg/kg/month) | 100  33  699 | 100  50  485 | -  0.63  0.05 |

CVID: common variable immunodeficiency; NRH: nodular regenerative hyperplasia; AIE: autoimmune enteropathy; AI: autoimmune; GLILD: granulomatous lymphocytic interstitial lung disease; HAV: hepatitis A virus; HBV: hepatitis B virus; HCV: hepatitis C virus; EBV: Epstein-Barr virus; CMV: cytomegalovirus; SCIG: subcutaneous immunoglobulin. Significance by chi-squared with Fisher’s exact test shown. Given that CVID patients receive passive antibody therapy, viral infection was defined as positive viral load by polymerase chain reaction only. Three CVID patients with NRH had low-grade (<100 IU/mL) EBV detection by PCR following NRH diagnosis. One CVID patient with NRH had low-grade CMV (<35 IU/mL) detection by PCR following NRH diagnosis.

**Supplemental Table 4. Comparison of liver parameters in CVID patients, with and without NRH, who underwent transient elastography.**

|  | **CVID with**  **NRH (N=12)** | **CVID without NRH (N=6)** | **p-value** |
| --- | --- | --- | --- |
| **Liver Biochemistries***  AST (U/L)  ALT (U/L)  ALP (U/L, proximal)  ALP (U/L, peak)  GGT** (U/L)  Albumin (g/dL)  Total Bilirubin (mg/dL)  PT/PTT (abnormal, %) | 54  44  250  314  151  4.3  0.75  25 | 26  26  100  114  518  3.9  0.35  0 | <0.01  0.15  0.03  0.02  0.18  0.02  0.01  0.51 |
| **Clinical Parameters of Portal Hypertension**  Splenic longitudinal diameter (cm, mean)  Upper limit normal (fold)  Varices (yes, %)  HVPG (mmHg, mean)  Ascites (yes, %)  Edema (yes, %)  Clinical portal hypertension (yes, %) | 16.6  1.5x  67  11.6  8  25  83 | 11.2  1x  0  -  0  0  0 | 0.02  -  0.04  -  1  0.51  <0.01 |

CVID: common variable immunodeficiency; NRH: nodular regenerative hyperplasia; AST: aspartate transaminase; ALT: alanine transaminase; ALP: alkaline phosphatase; GGT: gamma-glutamyl transferase; PT: prothrombin; PTT: partial thromboplastin; HVPG: hepatic venous pressure gradient. *Ammonia level not collected on any participants; ** γ-GGT, N=7 participants with data. Significance by one-way ANOVA.

**Supplemental Table 5. Controlled attenuation parameter by transient elastography is not significantly associated with clinical markers of portal hypertension.**

|  | **CAP (mean)** | **p-value** | **CAP (median)** | **p-value** |
| --- | --- | --- | --- | --- |
| **Clinical Parameters of Portal Hypertension**  Hepatic Venous Pressure Gradient*  >10 mmHg  </=10 mmHg    Varices  Yes  No  Ascites  Yes  No    Edema  Yes  No    Clinical Portal Hypertension  Yes  No | 227  183  248  169  228  197  267  184  210.0  186 | 0.33  0.1  0.64  0.22  0.52 | 223  176  233  136  228  219  267  188  224  181 | 1  0.22  0.32  0.13  1 |

CAP: controlled attenuation parameter (dB/m); *No difference in values using hepatic venous pressure gradient cutoff of 5 and 10 mmHg. Significance by one-way ANOVA and Mood’s median test.

**Supplemental Figure Legend.**

**Supplemental Figure 1. Correlation between controlled attenuation parameter and liver stiffness by transient elastography measurements.** Inclusive of patients with common variable immunodeficiency with controlled attenuation parameter available (N=10). Significance by spearman correlation coefficient. Line of best fit shown.
