# Supplementary figures and images for: Liver Stiffness by Transient Elastography Correlates With Degree of Portal Hypertension in Common Variable Immunodeficiency Patients With Nodular Regenerative Hyperplasia
Source: Front Immunol. 2022 May 6;13:864550. doi: 10.3389/fimmu.2022.864550 (PMC9121126; doi:10.3389/fimmu.2022.864550)

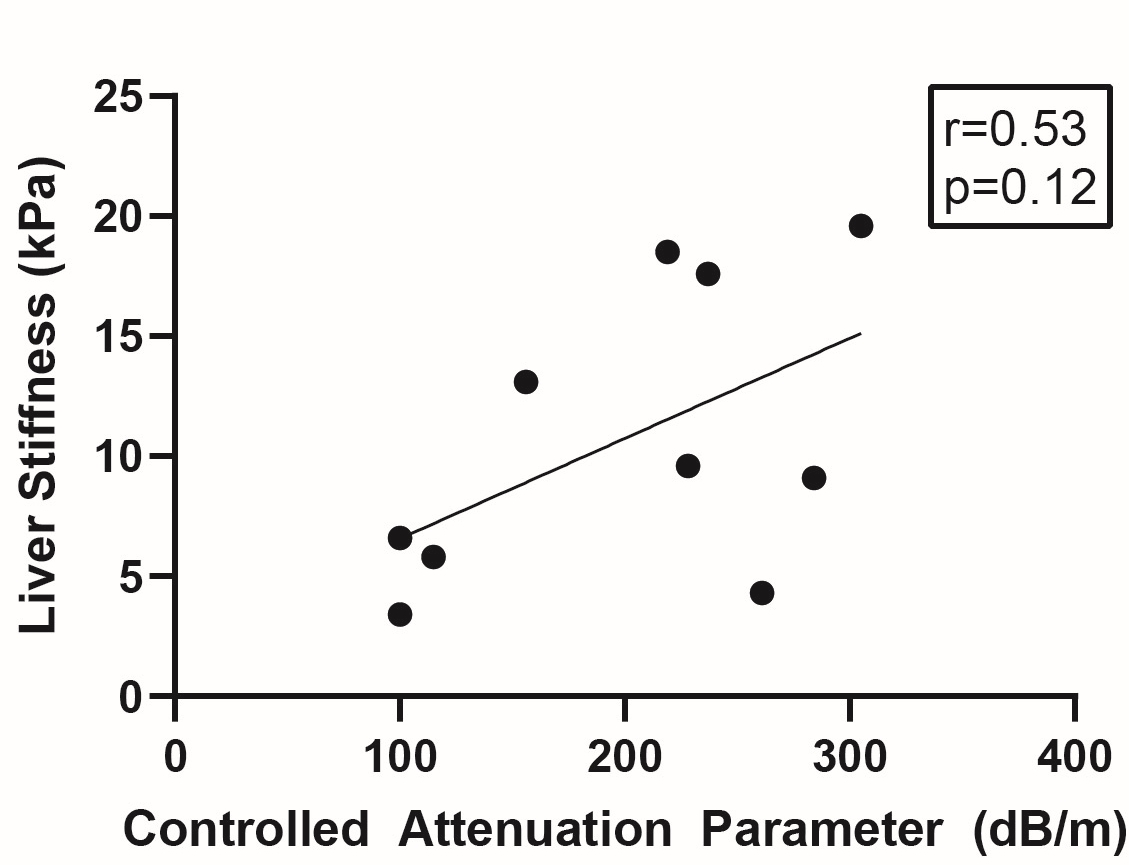

Supplement: Supplementary file 2 [file Image_1.jpg]
